# Primary Dermal Melanoma: A Rare Clinicopathological Variant Mimicking Metastatic Melanoma

**DOI:** 10.3390/dermatopathology8010005

**Published:** 2021-02-01

**Authors:** Oriana Simonetti, Elisa Molinelli, Valerio Brisigotti, Donatella Brancorsini, Davide Talevi, Annamaria Offidani

**Affiliations:** 1 Dermatological Unit, Department of Clinical and Molecular Sciences, Polytechnic Marche University, 60126 Ancona, Italy; molinelli.elisa@gmail.com (E.M.); valeriobrisigotti@hotmail.it (V.B.); annamaria.offidani@ospedaliriuniti.marche.it (A.O.); 2 Department of Biomedical Sciences, Institute of Pathological Anatomy and Histopathology, Polytechnic University Marche, 60126 Ancona, Italy; Donatella.Brancorsini@ospedaliriuniti.marche.it; 3 Reconstructive Plastic Surgery Unit, Department of Clinical and Molecular Sciences, Polytechnic Marche University, 60126 Ancona, Italy; Davide.Talevi@ospedaliriuniti.marche.it

**Keywords:** cutaneous melanoma, cutaneous metastatic melanoma, dermal melanoma, histopathologic diagnosis, primary dermal melanoma, prognosis

## Abstract

Primary dermal melanoma (PDM) is a rare distinct variant of cutaneous melanoma, predominantly occurring on the extremities of young or middle-aged adults. In comparison to conventional melanoma, PDM is characterized by unexpectedly prolonged survival and long-term survival. Thus, correct identification of this variant is crucial to avoid potential misdiagnosis and establish correct treatment and follow-up. In addition, no consensus and specific guidelines exist on the management of this peculiar subtype of cutaneous melanoma.

## 1. Introduction

Primary dermal melanoma (PDM) is a rare and distinct variant of cutaneous melanoma (CM), defined as a solitary dermal/subcutaneous nodule of melanoma and without an epidermal component. The most frequent clinical presentation is skin-colored to a bluish-red elevated firm nodule. Meanwhile, clinical differential diagnoses comprise nodular melanoma (NM), non-melanoma skin cancer (NMSC), blue nevus, hemangioma, dermatofibroma, cysts, and scars [1,2].

PDM histologically simulates cutaneous metastatic melanoma (CMM), from which it can often be indistinguishable. An excellent prognosis and long-term survival characterize it, compared with similarly staged NM or CMM (5-year survival rates of 73% to 100% in patients with PDM versus a survival rate of 52% at five years in patients with CMM of unknown primary origin) [3,4]. As the prognosis of PDM differs greatly from conventional CM, the distinction is crucial [4,5,6,7].

A 49-year-old man presented with a solitary, asymptomatic, slightly violaceous nodule (35 mm × 15 mm) on the medial part of the chest, exhibiting peripheral redness. The lesion had slowly developed during the last six years.

**Figure 1:**
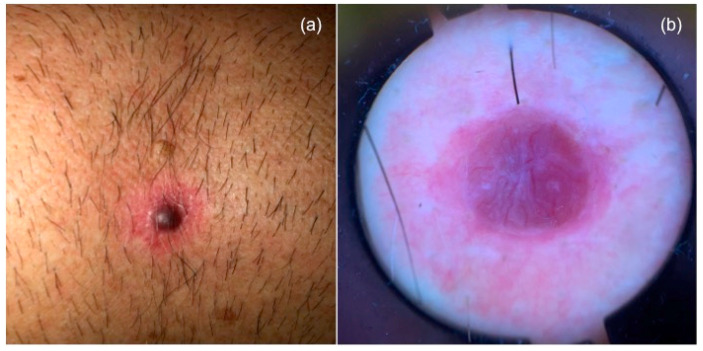
(**a**) Pink nodule with a peripheral erythematous halo of the chest. (**b**) Dermoscopic pattern of nodular lesion: reddish-purplish homogeneous pattern, with atypical vessels.

**Figure 2:**
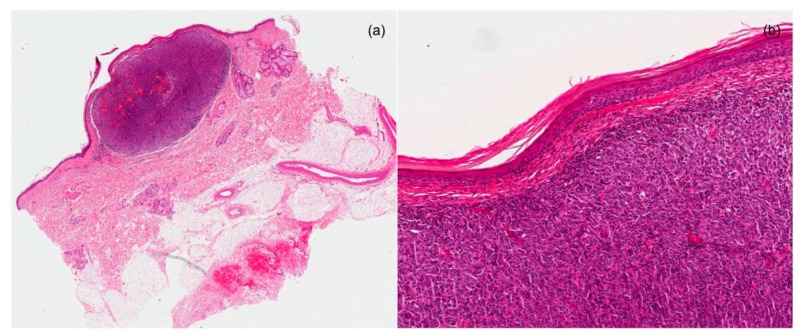
(**a**) Well-circumscribed dermal nodule with no involvement of the epidermis (EE × 2) (**b**) Proliferation of atypical and pleomorphic melanocytes with mitotic activity (EE × 200).

Dermoscopy revealed a reddish-purplish homogeneous pattern, with atypical vessels, mainly linear, irregular, and dotted. (Figure 1) The patient had no clinically palpable lymph node disease. Histopathological examination showed a circumscribed dermal-based melanocytic neoplasm (Breslow depth of 3.2 mm, Clark level IV, and mitotic rate 8/mm^2^). The melanocytes (epithelioid and spindled) were arranged either as single or multiple expansive nests. There was no evidence of any underlying in situ component, ulceration, blood or lymphatic vessel invasion, regression, or associated nevus. (Figure 2) Lymphocytic host response was evident at the periphery of the lesion. Immunohistochemical analysis showed strong immunostaining for S100 and a weaker tissutal expression of Ki67. Re-excision with clear margins from the primary melanoma was made. Metastatic staging workup findings were negative, including sentinel lymph node biopsy and full-body imaging studies with computed tomography and positron emission tomography. A definitive clinical and histological diagnosis of PDM was made. The patient did not receive any adjuvant treatment. No cutaneous and loco-regional recurrences or distant metastasis developed during 18 months of clinical and imaging (sonography/PET) follow up.

## 2. Discussion

PDM is a rare variant of CM, affecting less than 1% of patients with melanoma. It usually develops in male patients younger than 60 years old. Although the most common anatomic sites of involvement are the extremities, the trunk, head, and neck are also interesting [8].

The pathogenesis is largely unknown. PDM may arise from dermal melanocytes, embryologic-melanocytic migration remnants or aberrations, or melanocytes associated with appendageal structures in the dermis or subcutaneous tissue [9].

Histopathologic criteria for the diagnosis of PDM include dermal melanocytic neoplasm with nodular or multinodular architecture; features of malignancy such as cytological atypia, mitoses, area of necrosis; no evidence of an intraepidermal component (in situ); no ulceration that could compromise the identification of an intraepidermal component; positivity for S100; absence of continuity with peripheral nerves (to differentiate PDM from malignant neural tumors); absence of pre-existing nevus; and no regression [1,10,11].

Immunohistochemically, PDM is characterized by lower expression of p53, Ki-67, cyclin D1, and D2-40 expression compared with both CMM and NM [8,9,10]. However, significant histopathologic and immunohistochemical differences between PDM and CMM are frequently difficult to highlight. Thus, the clinical pathologic correlation is crucial for the correct diagnosis, and it is also based on the absence of any primary cutaneous or visceral melanoma, absence of a prior melanocytic nevus spontaneously regressed, and absence of lymph node, visceral, or central nervous system metastasis, at presentation [1,12].

The cases of putative PDM reported in the literature represent a heterogeneous and controversial group of melanomas, including primary nodular melanoma with an occult intraepidermal component, metastatic melanoma with an initial occult primary origin, melanoma arising within and obliterating a pre-existing melanocytic nevus, and a group of true PDM [13,14,15].

By definition, our reported case represents a true PDM owing to the absence of an in situ component, ulceration, overlying regression/scarring, and extracutaneous metastasis.

## 3. Conclusions

Dermatologists and pathologists should consider PDM in the differential diagnosis of patients with a solitary cutaneous melanoma mimicking metastasis of unknown origin. Familiarity with this subtype of melanoma is crucial to provide the diagnosis and delineate adequate management [2,14].

Moreover, most conventional staging parameters normally used for prognosis in CM have limited applicability to PDM. Breslow depth and ulceration have no statistically significant relationship with recurrence compared to conventional melanomas [3,4,15]. Additional research focusing on appropriate staging and outcome is necessary to delineate this largely indefinite melanoma variant better.
